# Polymorphisms in the selenoprotein S gene: lack of association with autoimmune inflammatory diseases

**DOI:** 10.1186/1471-2164-9-329

**Published:** 2008-07-14

**Authors:** Alfonso Martínez, Jose Luis Santiago, Jezabel Varadé, Ana Márquez, José Ramón Lamas, Juan Luis Mendoza, Hermenegildo de la Calle, Manuel Díaz-Rubio, Emilio G de la Concha, Benjamín Fernández-Gutiérrez, Elena Urcelay

**Affiliations:** 1 Immunology Department, Hospital Universitario San Carlos, Madrid, Spain; 2 Rheumatology Department, Hospital Universitario San Carlos, Madrid, Spain; 3 Gastroenterology Department, Hospital Universitario San Carlos, Madrid, Spain; 4 Endocrinology Department, Hospital Ramón y Cajal, Madrid, Spain

## Abstract

**Background:**

Selenoprotein S (SelS) protects the functional integrity of the endoplasmic reticulum against the deleterious effects of metabolic stress. *SEPS1/SelS *polymorphisms have been involved in the increased release of pro-inflammatory cytokines interleukin (IL)-1β, tumor necrosis factor (TNF)-α and IL-6 in macrophages. We aimed at investigating the role of the *SEPS1 *variants previously associated with higher plasma levels of these cytokines and of the *SEPS1 *haplotypes in the susceptibility to develop immune-mediated diseases characterized by an inflammatory component.

**Results:**

Six polymorphisms distributed through the *SEPS1 *gene (rs11327127, rs28665122, rs4965814, rs12917258, rs4965373 and rs2101171) were genotyped in more than two thousand patients suffering from type 1 diabetes, rheumatoid arthritis or inflammatory bowel diseases and 550 healthy controls included in the case-control study.

**Conclusion:**

Lack of association of *SEPS1 *polymorphisms or haplotypes precludes a major role of this gene increasing predisposition to these inflammatory diseases.

## Background

The human gene *SEPS1*, located on chromosome 15q26.3, encodes selenoprotein S which participates in the retro-translocation of misfolded proteins from the endoplasmic reticulum (ER) to the cytosol for their degradation [1]. This ER membrane protein functions in stress responses to prevent the deleterious consequences of accumulation of misfolded proteins, accumulation that has been linked to immune and inflammatory processes [2]. A study identified the strong association of the proximal promoter *SEPS1 *polymorphism at -105G/A with circulating levels of three pro-inflammatory cytokines, interleukin (IL)-6, IL-1β and TNF-α [3]. Moreover, these authors reported that the mutant variant significantly reduced the promoter activity of the *SEPS1 *gene in stressed HepG2 cells and that the suppression of this gene by short interfering RNA increased the release of pro-inflammatory cytokines in a macrophage cell line. A regulatory loop has been recently proposed whereby cytokines stimulate the expression of *SEPS1*, which in turn diminishes cytokine production [4].

The murine homolog gene of the human *SEPS1 *is the *Tanis *gene, which encodes a serum amyloid A receptor [5]. Acute phase serum amyloid A proteins (SAAs) are multifunctional apolipoproteins produced in large amounts during the acute phase of inflammation and also during the development of chronic inflammatory diseases. SAAs are involved in the pathogenesis of several chronic inflammatory diseases, such as rheumatoid arthritis (RA) [6-9], multiple sclerosis (MS)[10] and inflammatory bowel diseases (IBD) [11-13]. It is believed that locally synthesized SAA by synovial cells in the inflamed joints acts as an autocrine inducer of matrix metalloproteinase-1 and causes extensive joint erosion [14].

Altogether these data point to the important role of selenoprotein S mediating inflammation, and we aimed at testing whether the *SEPS1 *gene was involved in the development of inflammatory autoimmune complex diseases. These are multifactorial traits influenced by both genetic predisposing factors and environmental triggers. The activation of *SEPS1 *expression by fasting *in vivo *and by glucose-deprivation *in vitro *allowed its assignation to the glucose-regulated protein family [5,15]. Furthermore, a locus on 15q26, IDDM3, was described to increase susceptibility to type 1 diabetes, T1D [16,17]. Therefore, we decide to test the *SEPS1 *polymorphisms for association with diabetes risk in a cohort of Spanish T1D patients. Additionally, we pursued to study two other autoimmune diseases with an important inflammatory component, rheumatoid arthritis and the inflammatory bowel diseases, Crohn's disease (CD) and ulcerative colitis (UC). Provided this candidate gene previously related with the inflammatory response shows any influence on the pathogenesis of these diseases, a new mechanistic tool for treatment would be available.

## Methods

### Patients and controls

The study group consisted of 592 RA, 674 IBD and 311 T1D unrelated patients, consecutively recruited from one centre, either Hospital Clínico San Carlos or Hospital Ramón y Cajal (Madrid, Spain).

T1D patients (median age at onset 15 years) diagnosed according to the criteria of the American Diabetes Association (ADA), were insulin-dependent at the time to study.

RA diagnosis was established based on the American College of Rheumatology (ACR) criteria [18] and samples were previously genotyped for HLA-DRB1. Mean age at onset was 54 ± 14 years; 61% of the patients carried the shared epitope; 66% and 50% of the patients were positive for rheumatoid factor and for anti-cyclic citrullinated peptide, respectively, and 32% of the patients presented nodular disease.

Diagnosis of IBD patients was based on standard clinical, radiologic, endoscopic and histologic criteria [19]. The mean age at onset for UC patients was 38 years; 41% of the patients presented pancolitis; 47% and 13% of the patients suffered extraintestinal manifestations and colectomy, respectively. CD patients were classified according to the location of the lesions in ileal (L1, 48%), colonic (L2, 16%), ileocolonic (L3, 32%) and upper gastrointestinal tract (L4, 3%) and according to the disease behavior in inflammatory (B1, 43%), stricturing (B2, 15%) and perforating (B3, 42%). Only 20% of the CD patients debuted after the age of 40.

A group of 550 healthy unrelated subjects from Madrid (mainly hospital employees and blood donors) were selected as controls. Cases and controls were all white Spanish subjects and were included in this study after written informed consent. The Ethics Committee of Hospital Clínico (Madrid) approved the study.

### *SEPS1 *polymorphisms

The *SEPS1 *polymorphisms (rs11327127, rs28665122, rs4965814, rs12917258, rs4965373 and rs2101171) were genotyped using TaqMan assays from Applied Biosystems following manufacturer's suggestions and analyzed in an ABI 7900HT Fast Real-Time PCR System (Applied Biosystems, Foster City, CA, USA). Their location is indicated by the distance from the transcription start site: two of them are in the promoter region; another two in intron 5; rs4965373 depending of alternative splicing is either in intron 6 or in the 3'UTR region; and the last one is downstream at 9707 (see Table 1).

**Table 1:** Genotypic frequencies of the *SEL-S *polymorphisms in Spanish T1D, RA and IBD patients and controls.

	**T1D patients**	**RA patients**	**CD patients**	**UC patients**	**Controls**
***-538 SEPS1 *rs11327127**					
TT	183 (59%)	212 (59%)	218(62%)	235 (62%)	306 (60%)
T delT	106 (34%)	127 (35%)	111 (32%)	124 (32%)	180 (35%)
delT delT	21 (7%)	20 (6%)	21 (6%)	22 (6%)	28 (5%)
***-105 SEPS1 *rs28665122**					
CC	216 (70%)	253 (70%)	253(73%)	274 (71%)	360 (72%)
CT	85 (27%)	92 (26%)	83 (24%)	100 (26%)	124 (25%)
TT	9 (3%)	14 (4%)	12 (3%)	11 (3%)	14 (3%)
***SEPS1 3705 *rs4965814**					
TT	195 (63%)	224 (62%)	215 (68%)	225 (64%)	334 (65%)
TC	103 (33%)	118 (33%)	82 (26%)	112 (32%)	161 (31%)
CC	12 (4%)	17 (5%)	19 (6%)	12 (4%)	20 (4%)
***SEPS1 4283 *rs12917258**					
CC	148 (48%)	150 (42%)	164 (46%)	165 (44%)	215 (42%)
CG	137 (44%)	163 (45%)	149 (42%)	173 (46%)	247 (48%)
GG	25 (8%)	46 (13%)	40 (11%)	38 (10%)	49 (10%)
***SEPS1 5227 *rs4965373**					
GG	145 (47%)	187 (52%)	182 (52%)	178 (48%)	246 (48%)
GA	127 (41%)	142 (40%)	136 (39%)	163 (43%)	220 (43%)
AA	38 (12%)	30 (8%)	33 (9%)	33 (9%)	43 (9%)
***SEPS1 9707 *rs2101171**					
TT	177 (57%)	218 (61%)	216 (61%)	223 (59%)	296 (58%)
TC	113 (37%)	130 (36%)	122 (34%)	138 (37%)	191 (37%)
CC	20 (6%)	11 (3%)	16 (5%)	17 (4%)	26 (5%)

### Statistical Analysis

Allele and genotype frequencies in patients and controls were compared by χ^2 ^test or Fisher exact test when necessary; p values were considered significant at a level of <0.05. Odds ratio (OR) and p values were calculated using a standard package (Epi Info v. 6.02, CDC, Atlanta, USA). For an OR = 1.5, the statistical power of our cohorts ranged from 80 – 95% depending on the specific polymorphism analyzed.

Haplotypic frequencies were estimated using the Expectation-Maximization algorithm implemented in the Arlequin v2.000 software [20] with number of iterations set at 5000 and initial conditions at 50, with an epsilon value of 10^-7^.

## Results and discussion

The present study investigates the influence of all *SEPS1 *variants previously correlated with increased pro-inflammatory cytokines levels (polymorphisms at -105G/A, 3705G/A and 5227C/T) and of the *SEPS1 *haplotypes on predisposition to inflammatory complex diseases.

We first analyzed the functional *SEPS1 *promoter polymorphism at -105G/A and no significant differences in genotypic frequencies were observed when healthy controls were compared with patients suffering from T1D, RA or IBD (Table 1). As two other variants (rs4965814 and rs4965373) have been associated with high plasma cytokine levels [3], we studied them in our cohorts, and the distribution of genotypes followed the same pattern in patients and in controls (Table 1).

We selected three additional polymorphisms along the gene from those described by Curran et al [3] in order to analyze the haplotypes inferred by applying the Expectation-Maximization algorithm implemented in the Arlequin software. No significant difference in haplotype frequencies was observed after appropriate correction for multiple testing in any diseased cohort (Table 2).

**Table 2:** Haplotypes estimated within the *SEPS1 *gene (alleles at -538/-105/3705/4283/5227/9707) and their distribution in patients and healthy individuals.

	**T1D (2n = 620)**		**RA (2n = 718)**		**CD (2n = 582)**		**UC (2n = 664)**		**Controls (2n = 982)**	
	
	**Freq.**	**HT**	**Freq.**	**HT**	**Freq.**	**HT**	**Freq.**	**HT**	**Freq.**	**HT**
**TCTGGT**	0.28827	179	0.32557	234	0.334699	195	0.339168	225	0.325571	320
**TCTCGT**	0.1716	106	0.1711	123	0.185524	108	0.176243	117	0.171104	168
**TCTCAC**	0.16575	103	0.15691	113	0.140554	82	0.161364	107	0.156905	154
**DelT TCCGT**	0.13375	83	0.12057	87	0.124047	72	0.122667	81	0.120574	118
**DelT CTCAC**	0.06887	43	0.07503	54	0.063144	37	0.051819	34	0.075028	74
**TCTCAT**	0.07046	44	0.05216	37	0.069939	41	0.060517	40	0.052158	51
**TCCCGT**	0.02949	18	0.02635	20	0.027632	16	0.027826	18	0.026349	26
**TTCCGT**	0.01231	8	0.01671	12	0.011366	7	0.010658	7	0.018029	18
**DelT CCCGT**	0.00875	5	0.01114	8	0.019499	11	0.015959	11	0.015536	15
**Others**	0.05075	31	0.04178	30	0.023596	13	0.033779	24	0.038746	38

A direct functionality has been attributed to the *SEPS1 *promoter polymorphism at -105G/A, increasing plasma levels of the aforementioned cytokines presumably due to the disruption of an ER stress element (ERSE). Two other tested variants have also been strongly related with the circulating levels of the inflammatory cytokines [3]. In contrast, a recent report did not find consistent association of this polymorphism with circulating levels of either IL-6 or TNF-α [21]. We did not observe differences in the genotypic distribution of the *SEPS1 *polymorphisms analyzed in Spanish cohorts between patients suffering from autoimmune diseases with a clear inflammatory component, like T1D, RA or IBD, and controls. The analysis of *SEPS1 *haplotypic frequencies did not differ between patients and healthy individuals either.

Given that evidences exist for the affected expression and activity of some selenoproteins depending on sexual dimorphism in mouse [22] and human [23], we decided to check for a gender specific association of -105G/A *SEPS1 *polymorphism with the autoimmune disorders under study; however, we did not detect a gender-bias in our results. No significant difference between sex-stratified cohorts was found for any of the polymorphisms studied (Table 3).

**Table 3:** Genotype frequencies of *SEPS1 *polymorphisms in Spanish patients stratified by gender.

	**T1D Women (n, %)**	**T1D Men (n, %)**	**RA Women (n, %)**	**RA Men (n, %)**	**CD Women (n, %)**	**CD Men (n, %)**	**UC Women (n, %)**	**UC Men (n, %)**
***-538 SEPS1 *rs11327127**								
TT	90 (58)	93 (60)	160 (59)	49 (60)	108 (60)	109 (64)	97 (64)	135 (60)
T delT	53 (34)	53 (34)	100 (37)	24 (30)	61 (34)	50 (30)	49 (33)	72 (32)
delT delT	12 (8)	9 (6)	12 (4)	8 (10)	10 (6)	10 (6)	4 (3)	18 (8)
***-105 SEPS1 *rs28665122**								
CC	107 (69)	109 (70)	191 (70)	58 (71)	128 (72)	123 (73)	106 (71)	160 (71)
CT	43 (28)	42 (27)	70 (26)	20 (25)	44 (25)	38 (23)	42 (28)	56 (25)
TT	5 (3)	4 (3)	11 (4)	3 (4)	5 (3)	7 (4)	2 (1)	9 (4)
***SEPS1 3705 *rs4965814**								
TT	99 (64)	96 (62)	172 (63)	49 (60)	109 (68)	104 (68)	92 (69)	129 (62)
TC	50 (32)	53 (34)	87 (32)	28 (35)	43 (27)	40 (26)	38 (29)	70 (34)
CC	6 (4)	6 (4)	13 (5)	4 (5)	8 (5)	10 (6)	3 (2)	9 (4)
***SEPS1 4283 *rs12917258**								
CC	82 (53)	66 (43)	109 (40)	39 (48)	78 (43)	84 (50)	69 (47)	92 (41)
CG	61 (39)	76 (49)	124 (46)	35 (43)	85 (47)	63 (37)	59 (40)	113 (51)
GG	12 (8)	13 (8)	39 (14)	7 (9)	17 (9)	23 (13)	20 (13)	18 (8)
***SEPS1 5227 *rs4965373**								
GG	71 (46)	74 (48)	150 (55)	34 (42)	92 (51)	89 (53)	74 (51)	102 (46)
GA	61 (39)	66 (42)	103 (38)	37 (45)	72 (40)	62 (37)	51 (35)	105 (48)
AA	23 (15)	15 (10)	19 (7)	10 (12)	16 (9)	17 (10)	20 (14)	13 (6)
***SEPS1 9707 *rs2101171**								
TT	87 (56)	90 (58)	172 (63)	43 (53)	111 (61)	105 (61)	96 (64)	124 (55)
TC	55 (36)	58 (37)	91 (34)	36 (44)	64 (35)	56 (33)	40 (27)	95 (43)
CC	13 (8)	7 (5)	9 (3)	2 (3)	6 (3)	10 (6)	13 (9)	4 (2)

*SEPS1 *expression was not significantly altered in intestinal epithelial cells of IBD murine models or in intestinal biopsies from IBD patients when compared with that present in controls [24]. Concordantly, a study testing the association of the promoter variant at -105 with cerebrovascular disease found no influence on stroke risk [25]. Both studies are in agreement with our results that showed lack of association between *SEPS1 *polymorphisms and susceptibility to chronic inflammatory diseases. Moreover, a Finnish study of five variants in the *SEPS1 *gene locus showed no significant difference between carriers/non-carriers when cardiovascular cases and healthy individuals were compared. The authors suggested that two polymorphisms contribute to the risk for coronary heart disease and for ischemic stroke in females; interestingly, the associated polymorphisms do not correspond with the one at -105G/A [21]. Therefore, evidences are mounting against the role of the *SEPS1 *gene in all these conditions, with the only exception to this point of one report defining the minor allele at -105G/A *SEPS1 *as a risk factor for preeclampsia in a large Norwegian cohort [26].

## Conclusion

The Wellcome Trust Case Control Consortium published recently a genome wide association study for several inflammatory conditions, such as RA, T1D or CD [27] Although the chromosomal region where the gene maps did not show association for any of the mentioned diseases, the *SEPS1 *polymorphisms were not analyzed and therefore their involvement in susceptibility could not be formally excluded. Ours is a thorough study analyzing the influence of six polymorphisms along the *SEPS1 *gene on susceptibility to common diseases, unravelling lack of association. Our data allow discardind a major individual role of this gene in the aetiology of the studied polygenic diseases.

## Abbreviations

***SelS(SEPS1)***: Selenoprotein S gene; **(IL)-1β**: Interleukin 1 beta; **TNF-α**: Tumor necrosis factor alpha; **IL-6**: Interleukin 6; **ER**: Endoplasmic reticulum; **HepG2**: Human hepatocellular liver carcinoma cell line; **RNA**: Ribonucleic acid; **SAAs**: Acute phase serum amyloid A proteins; **RA**: Rheumatoid arthritis; **MS**: Multiple sclerosis; **IBD**: Inflammatory bowel diseases; **IDDM3**: Insulin-dependent diabetes mellitus 3; **T1D**: Type 1 diabetes; **CD**: Crohn's disease; **UC**: Ulcerative colitis; **ERSE**: ER stress element; **ADA**: American Diabetes Association; **ACR**: American College of Rheumatology; **L1**: Ileal lesions; **L2**: Colonic lesions; **L3**: Ileocolonic lesions; **L4**: Upper gastrointestinal tract lesions; **B1**: Inflammatory disease behavior; **B2**: Structuring disease behavior; **B3**: Perforating disease behavior; **OR**: Odds ratio; **FIS**: Fondo de Investigaciones Sanitarias.

## Authors' contributions

JLS, JV and AnM, carried out the genotyping of the patients and a great part of the controls, participated in the statistical analysis and drafted the manuscript. JLM, HdlC, MDR, JRL and BFG made the diagnosis and collaborated in collection of samples. AM participated in the coordination of the study and participated in the statistical analysis. EGdlC coordinated the study and critically revised the manuscript. EU conceived of the study, participated in the statistical analysis and completed the writing of the manuscript. All authors read and approved the final manuscript.
